# Cortical Inhibition State-Dependent iTBS Induced Neural Plasticity

**DOI:** 10.3389/fnins.2022.788538

**Published:** 2022-02-17

**Authors:** Xiaoying Diao, Qian Lu, Lei Qiao, Youhui Gong, Xiao Lu, Min Feng, Panpan Su, Ying Shen, Ti-Fei Yuan, Chuan He

**Affiliations:** ^1^Department of Rehabilitation Medicine, The Affiliated Jiangsu Shengze Hospital of Nanjing Medical University, Suzhou, China; ^2^Shanghai Key Laboratory of Psychotic Disorders, Shanghai Mental Health Center, Shanghai Jiao Tong University School of Medicine, Shanghai, China; ^3^Jiangsu Zhongshan Geriatric Rehabilitation Hospital, Nanjing, China; ^4^Rehabilitation Medicine Center, The First Affiliated Hospital of Nanjing Medical University, Nanjing, China; ^5^Co-innovation Center of Neuroregeneration, Nantong University, Nantong, China; ^6^Translational Research Institute of Brain and Brain-Like Intelligence, Shanghai Fourth People’s Hospital Affiliated to Tongji University School of Medicine, Shanghai, China

**Keywords:** intermittent theta burst stimulation, LTP-like plasticity, GABA_A_ receptor-mediated activity, GABA_B_ receptor-mediated activity, *N*-methyl-D-aspartate receptor-mediated activity

## Abstract

**Background:**

Intermittent theta burst stimulation (iTBS) is an effective stimulus for long-term potentiation (LTP)-like plasticity. However, iTBS-induced effects varied greatly between individuals. Ample evidence suggested that an initial decrease in local γ-aminobutyric acid (GABA) or enhancement in *N*-methyl-D-aspartate (NMDA) facilitation neurotransmission is of vital importance for allowing LTP-like plasticity to occur. Therefore, we aimed to investigate whether the individual level of GABA or NMDA receptor-mediated activity before stimulation is correlated with the after-effect in cortical excitability induced by iTBS.

**Methods:**

Fifteen healthy volunteers were recruited for the present study. We measured short-interval intracortical inhibitory (SICI), long-interval intracortical inhibitory (LICI), and intracortical facilitation (ICF), which index GABA_A_ receptor-, GABA_B_ receptor-, and glutamate receptor-mediated activity, respectively, in the cortex before conducting iTBS. After iTBS intervention, the changes of motor-evoked potential (MEP) amplitude were taken as a measure for cortical excitability in response to iTBS protocol.

**Results:**

There was a significant negative correlation between the amount of SICI measured before iTBS and the after-effect of iTBS-induced LTP-like plasticity at the time points of 5, 10, and 15 min after inducing iTBS. A multiple linear regression model indicated that SICI was a good predictor of the after-effect in cortical excitability induced by iTBS at 5, 10, and 15 min following stimulation.

**Conclusion:**

The present study found that GABA_A_ receptor-mediated activity measured before stimulation is negatively correlated with the after-effect of cortical excitability induced by iTBS. SICI, as the index of GABA_A_ receptor-mediated activity measured before stimulation, might be a good predictor of iTBS-induced LTP-like plasticity for a period lasting 15 min following stimulation.

## Introduction

Long-term potentiation (LTP)-like plasticity, defined as the ability of neurons to activity-dependently modify the strength of synaptic transmission, is the most common form of synaptic plasticity (Hebb, 2005; Takeuchi et al., 2014; Llona et al., 2017; Mateos-Aparicio and Rodríguez-Moreno, 2019). It is significant in response to physiological degeneration or brain injury (Wieloch and Nikolich, 2006; Chen et al., 2010; Cramer et al., 2011). LTP has been found to be induced by repetitive electrical stimulation in animal experiments, but recently the introduction of transcranial magnetic stimulation (TMS) with protocols of repetitive TMS (rTMS) presented the possibility of delivering similar LTP-like plasticity in the human brain (Bliss and Lømo, 1973; Wang et al., 1996; Ziemann et al., 1998b; Siebner and Rothwell, 2003; Cooke, 2006). Therefore, these rTMS-induced synaptic changes might have significant implications for therapeutic opportunities after brain damage *via* mechanisms of cortical plasticity (Lefaucheur et al., 2020).

Intermittent theta burst stimulation (iTBS) is one such protocol that can result in increases of cortical excitability persisting beyond the period of stimulation (Huang et al., 2005; Di Lazzaro et al., 2008). Compared with traditional rTMS protocols, iTBS requires lower simulation intensity and less stimulation time for inducing similar after-effects (Todd et al., 2009). Although iTBS may be indicative of an appealing technique for modulating cortical plasticity for clinical or therapeutic applications, recent studies observed that the effect varies greatly between individuals (Hamada et al., 2013; Hinder et al., 2014; Schilberg et al., 2017). Ridding and Ziemann (2010) summarized that the interindividual variability may depend on several different factors such as age, genetics, pharmacological influences, and neural activity in the brain before conducting stimulation. Such variability at present limits the therapeutic effectiveness of iTBS for inducing plastic changes.

The present study aimed at testing one such important factor that contributes to the variation of iTBS-induced plastic changes. Accumulating evidence in animal studies suggested that susceptibility to cortical potential-like plasticity is influenced by the level of cortical NMDAergic excitability and GABAergic inhibition (Kano and Iino, 1991; Schwenkreis et al., 2003; Di Lazzaro et al., 2006; Bachtiar and Stagg, 2014). Hess et al. (1996) suggested that LTP was enhanced by blockade of GABA_A_ receptors with antagonist bicuculline in the motor cortex; conversely, the *N*-methyl-D-aspartate (NMDA) antagonist 2-amino-5-phosphonovaleric acid blocked LTP induction. LTP has also been found to be induced through iTBS when GABA_A_ and GABA_B_ receptors were both blocked (Kotak et al., 2017).

In human studies, paired pulse transcranial magnetic stimulation (ppTMS) can be performed to evaluate the level of GABA or NMDA receptor-mediated activity (Kujirai et al., 1993; Ziemann et al., 1998a; Ilić et al., 2002; McDonnell et al., 2006; Rossini et al., 2015). Therefore, we wished to verify whether the after-effect in cortical excitability induced by iTBS is correlated with the level of GABA or NMDA receptor-mediated activity before stimulation. Here, ppTMS was performed to evaluate short-interval intracortical inhibitory (SICI), long-interval intracortical inhibitory (LICI), and intracortical facilitation (ICF), which index GABA_A_ receptor-, GABA_B_ receptor-, and glutamate receptor-mediated activity, respectively.

## Materials and Methods

### Participants

Fifteen healthy volunteers (13 females) were recruited for the present study. Age ranges from 20 to 23 years (*M* = 21.07, *SD* = 1.06). All participants were right-handed (assessed by the Edinburgh Handedness Inventory; Oldfield, 1971) and had normal or correlated-with-normal vision. Exclusion criteria included a history of psychiatric or neurologic diseases, epilepsy, cardiovascular complications, taking any medication on a regular basis, and contraindications to TMS (e.g., taking epileptogenic drugs, implants in the brain, pregnant women). Informed consent was obtained from all participants. The study was performed according to the Declaration of Helsinki and approved by the Ethics Committee of Affiliated Jiangsu Shengze Hospital of Nanjing Medical University (JSSZYY-LLSC-202104). The study was registered with the China Clinical Trial Registration Center^1^ under the number ChiCTR2100046794.

### Transcranial Magnetic Stimulation and Electromyography Recordings

**FIGURE 1:**
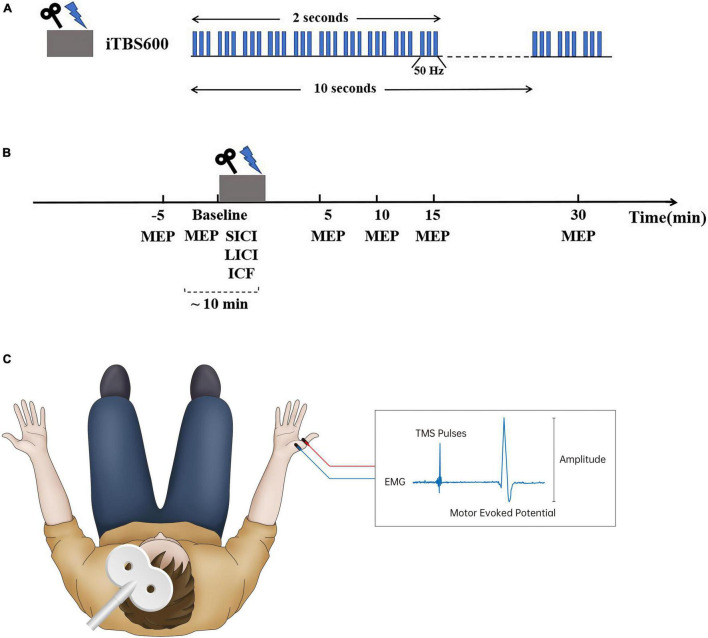
Experimental design. **(A)** Intermittent theta burst stimulation (iTBS) protocol consisted of bursts containing three pulses given at 50 Hz and repeated every 200 ms; a 2-s train of this stimulating pattern was repeated every 10 s for a total of 190 s (600 pulses). **(B)** Each subject was assessed motor-evoked potentials (MEPs) and short-interval intracortical inhibitory (SICI), long-interval intracortical inhibitory (LICI), and intracortical facilitation (ICF) successively before iTBS intervention. Following iTBS, MEPs were assessed at 5, 10, 15, and 30 min after stimulation. **(C)** Transcranial magnetic stimulation (TMS) was performed over the hand region of the left primary motor cortex (LM1) connected with a figure-of-eight coil. The stimulating coil was placed tangentially to the scalp with the handle pointing posteriorly and laterally 45° to the sagittal plane over the LM1 region. MEPs were recorded from the right abductor pollicis brevis (APB) muscle by use of silver/carbon-backed electrodes placed 2 cm apart in a belly-tendon montage to assess cortical excitability.

Single monophasic TMS was performed over the hand region of the left primary motor cortex (LM1) using the Neuro-MS/D stimulator (Neurosoft Llc, Ivanovo, Russia) connected with a figure-of-eight coil (external loop diameters, 70 mm; peak magnetic field, 4 Tesla). The optimal coil position was determined by moving the coil in 1-cm steps around the presumed left M1 of hand until the point of the largest motor-evoked potential (MEP) amplitude of the relaxed abductor pollicis brevis (APB) muscle was reached. The stimulating coil was placed tangentially to the scalp with the handle pointing posteriorly and laterally 45° to the sagittal plane over the LM1 region. The stimulation intensity was determined in relation to the resting motor threshold (RMT) which was defined as the minimum TMS intensity eliciting a peak-to-peak MEP-amplitude of 50 μV or more in resting muscle, in at least 5 out or 10 trials (Groppa et al., 2012; Rossini et al., 2015). To assess the motor cortex excitability, motor-evoked potentials (MEPs) were recorded from the right APB muscle at rest (dominant hand in all participants) by use of silver/carbon-backed electrodes Skintact RT-34 (Fannin Ltd., Dublin, Ireland) with the size of 10.5 mm × 25 mm placed 2 cm apart in a belly-tendon montage (see Figure 1). The Neuro-MEP-Micro software was used to measure the amplitude of MEPs (Neurosoft Llc, Ivanovo, Russia).

### Intermittent Theta Burst Stimulation

iTBS was delivered over the hotspot of the LM1 using the Neuro-MS/D stimulator (Neurosoft Llc, Ivanovo, Russia). The stimulating protocol was conducted in accordance with the protocol originally described by Huang et al. (2005), which consisted of bursts containing three pulses given at 50 Hz and repeated every 200 ms (see Figure 1A). A 2-s train of this stimulating pattern was repeated every 10 s for a total of 190 s (600 pulses) (Huang et al., 2005). The stimulation intensity was set at 80% of RMT (Bulteau et al., 2017; Fujiki et al., 2020).

### Experimental Procedure

Participants were seated in a comfortable chair with a neck support and were asked to relax their right arm entirely. They were also continually reminded to keep their eyes open and fixate forward on throughout each trial. Eyes open and muscle relaxation were observed by visual or electromyography (EMG) monitoring. A hotspot was marked as the optimal coil position where single-pulse TMS produced the largest MEP amplitude of the relaxed APB muscle by moving the coil around the presumed left M1 of hand (Rossini et al., 2015). Single-pulse TMS with intensity of 120% RMT was conducted to assess the excitability of the corticospinal system before and after iTBS. The peak-to-peak amplitude of MEPs evoked by a suprathreshold stimulus with an intensity of 120% RMT was used to probe the excitability of the motor cortex.

Before conducting iTBS, each participant received two sessions of single-pulse TMS with 20 consecutive pulses of each with an interval of 5 min at baseline to confirm intraindividual reliability of cortical excitability; the following trials could begin until the difference between average MEPs in two sessions of measurement was no more than 20% (Yu et al., 2020). To quantify the level of GABA or NMDA receptor-mediated activity before stimulation, we measured SICI, LICI, and ICF successively before conducting iTBS using a paired pulse paradigm at rest (Kujirai et al., 1993) (see Figure 1). SICI and ICF were delivered with an intensity of 90% RMT for the conditioning stimulus (CS) and 120% RMT for the testing stimulus (TS), with an interstimulus interval (ISI) of 2.5 and 12 ms, respectively, for 10 consecutive trials (Ozdemir et al., 2017; Tran et al., 2020). The CS and TS delivered in LICI were both set at 120% RMT, with an ISI of 150 ms for 10 consecutive trials (Mancheva et al., 2017). Paired pulse TMS results were based on 10 trials with single pulses (unconditioned) and 10 trials with paired pulses (conditioned) as previously recommended (Rossini et al., 2015).

### Statistical Analysis

All analyses were performed using IBM SPSS version 22 (Armonk, NY, United States), and statistical significance was set at *p* < 0.05. Data were first tested to evaluate the normal distribution using the Shapiro–Wilk test. The MEPs were normalized to baseline MEP amplitude for each participant to calculate the after-effect of iTBS-induced LTP-like plasticity. Paired-pulse TMS protocols were expressed as the ratio of conditioned MEPs to unconditioned MEPs. A one-way within-subject ANOVA was conducted on the LTP-like after-effect induced by iTBS among different time points (baseline, 5, 10, 15, 30 min). The Mauchly test was used to verify the sphericity. A two-sided Pearson correlation test was used to examine relationships between SICI/LICI/ICF measured before stimulation and the after-effect of iTBS-induced LTP-like plasticity at 5, 10, 15, and 30 min after conducting iTBS, respectively. Multiple tests were corrected using the false discovery rate (FDR) method (Benjamini and Yekutieli, 2001) for ANOVA and Pearson correlation. To examine whether iTBS-induced plasticity can be predicted by SICI, LICI, or ICF, we also performed multiple regression analysis using the stepwise method. The multicollinearity test was performed based on the variance inflation factor (VIF) to examine whether our data met the assumption of collinearity.

## Results

### Intermittent Theta Burst Stimulation-Induced Plasticity

**FIGURE 2:**
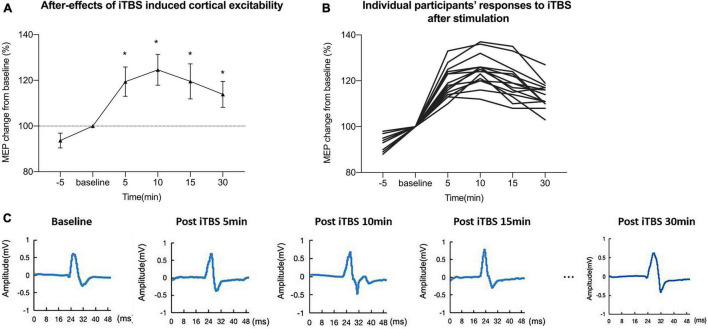
Intermittent theta burst stimulation (iTBS) induced increase in cortical excitability at different time points. **(A)** iTBS induced a mean increase in motor-evoked potential (MEP) amplitude at different time points after intervention. **(B)** Individual responses to iTBS after stimulation of each participant. **(C)** Representative traces in motor-evoked potentials (MEPs) recorded at 5, 10, 15, and 30 min after stimulation. The data indicated that the MEP amplitude began to increase after conducting iTBS.

Figure 2 shows an iTBS-induced increase in cortical excitability at different time points after inducing iTBS and individual participants’ responses to iTBS. Figure 2C shows representative changes in MEPs recorded at 5, 10, 15, and 30 min after stimulation. The data indicated that the MEP amplitude began to increase after conducting iTBS. A one-way within-subject ANOVA was conducted on the LTP-like after-effect induced by iTBS. There was a significant effect of the after-effect of iTBS-induced LTP-like plasticity among different time points (baseline, 5, 10, 15, 30 min): *F*(4,56) = 123.9, *p* < 0.001 (FDR corrected), η_*p*_2 = 0.87.

### After-Effect of Intermittent Theta Burst Stimulation-Induced Potential-Like Plasticity Correlates With the Level of Short-Interval Intracortical Inhibitory Before Stimulation

**FIGURE 3:**
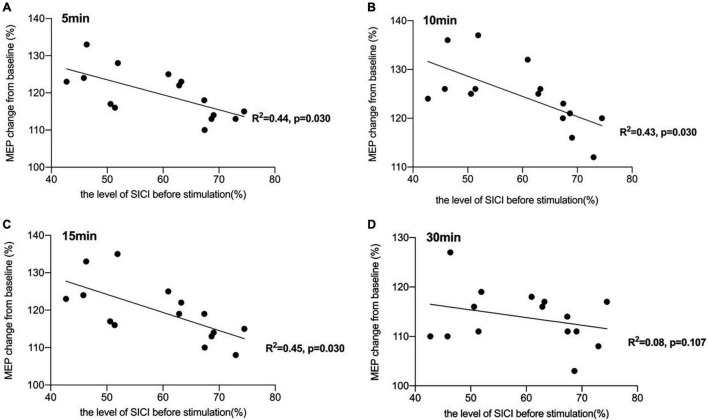
Correlation between short-interval intracortical inhibitory (SICI) and the after-effect of intermittent theta burst stimulation (iTBS)-induced long-term potentiation (LTP)-like plasticity. **(A)** There was a significant negative correlation between the amount of SICI measured before iTBS and the after-effect of iTBS-induced LTP-like plasticity at the time point of 5 min [*R^2^* = 0.44, *p* < 0.05, false discovery rate (FDR) corrected]. **(B)** The correlation between the level of SICI before stimulation and iTBS-induced after-effects at 10 min after conducting iTBS was also significant (*R^2^* = 0.43, *p* < 0.05, FDR corrected). **(C)** A significant negative correlation was also reported between the amount of SICI before stimulation and the iTBS-induced effects at 15 min after stimulation (*R^2^* = 0.45, *p* < 0.05, FDR corrected). **(D)** There was no significant correlation between SICI before stimulation and iTBS effect at 30 min (*R^2^* = 0.08, *p* = 0.107, FDR corrected).

**FIGURE 4:**
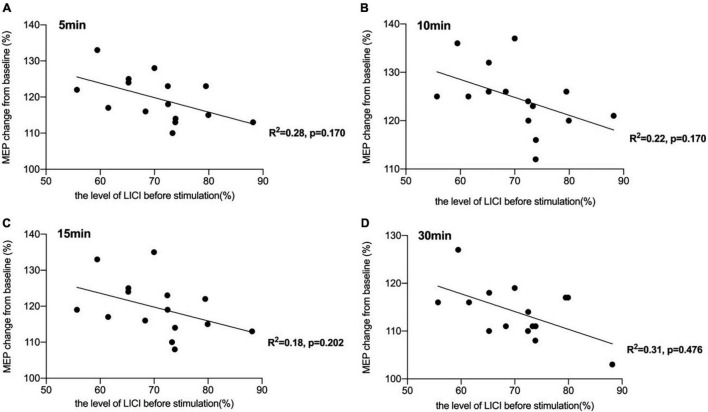
Correlation between long-interval intracortical inhibitory (LICI) and the after-effect of intermittent theta burst stimulation (iTBS)-induced long-term potentiation (LTP)-like plasticity. **(A)** No significant correlation was reported between the amount of LICI measured before iTBS and the iTBS after-effect at the time point of 5 min [*R^2^* = 0.28, *p* = 0.170, false discovery rate (FDR) corrected]. **(B)** No significant correlation was also reported between the amount of LICI before stimulation and the iTBS-induced effects at 10 min after stimulation (*R^2^* = 0.22, *p* = 0.170, FDR corrected). **(C)** The correlation between the level of LICI before stimulation and iTBS-induced after-effects at 15 min after conducting iTBS was also not significant (*R^2^* = 0.18, *p* = 0.202, FDR corrected). **(D)** There was also no significant correlation between the level of LICI and the after-effect of iTBS-induced LTP-like plasticity at the time point of 30 min (*R^2^* = 0.31, *p* = 0.476, FDR corrected).

**FIGURE 5:**
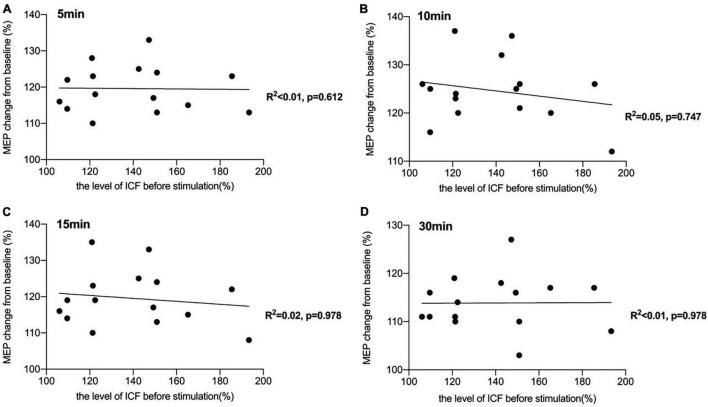
Correlation between intracortical facilitation (ICF) and the after-effect of intermittent theta burst stimulation (iTBS)-induced long-term potentiation (LTP)-like plasticity. **(A)** No significant correlation was reported between the amount of ICF measured before iTBS and the iTBS after-effect at the time point of 5 min [*R^2^* < 0.01, *p* = 0.612, false discovery rate (FDR) corrected]. **(B)** There was also no significant correlation between the level of ICF and the after-effect of iTBS-induced LTP-like plasticity at the time point of 10 min (*R^2^* = 0.05, *p* = 0.747, FDR corrected). **(C)** A non-significant correlation was also reported between the amount of ICF before stimulation and the iTBS-induced effects at 15 min after stimulation (*R^2^* = 0.02, *p* = 0.978, FDR corrected). **(D)** There was no significant correlation between ICF before stimulation and iTBS-effect at 30 min (*R^2^* < 0.01, *p* = 0.978, FDR corrected).

There was a significant negative correlation between the amount of SICI measured before iTBS and the after-effect of iTBS-induced LTP-like plasticity at the time points of 5 min (*R^2^* = 0.44, *p* < 0.05, FDR corrected), 10 min (*R^2^* = 0.43, *p* < 0.05, FDR corrected), and 15 min (*R^2^* = 0.45, *p* < 0.05, FDR corrected) following conduction of iTBS (Figure 3). *Post hoc* power analysis indicated that the power to detect the observed effects at the 0.05 level was 0.742, 0.760, and 0.807 at the time points of 5, 10, and 15 min, respectively, while there is no significant correlation between SICI before stimulation and iTBS effect at 30 min (*R^2^* = 0.08, *p* = 0.107, FDR corrected). No correlation between iTBS after-effect and the level of LICI was found at all time points 5, 10, 15, and 30 min after stimulation (Figure 4). Similarly, the iTBS after-effect was not correlated with the level of ICF measured before iTBS at all time points (Figure 5). Table 1 shows all *R*^2^ and *p*-values before and after applying the FDR correction.

**TABLE 1:** Correlation between the level of short-interval intracortical inhibitory (SICI), long-interval intracortical inhibitory (LICI), and intracortical facilitation (ICF) measured before stimulation and the after-effect of intermittent theta burst stimulation (iTBS)-induced long-term potentiation (LTP)-like plasticity (all *R*^2^ and *p*-values before and after applying the FDR correction).

ppTMS protocol	Time points after iTBS (min)	R^2^	*p*-value before FDR correction	*p*-value after FDR correction	Results after FDR correction
SICI	5	0.44	0.005	0.030	Significant
	10	0.43	0.006	0.030	Significant
	15	0.45	0.008	0.030	Significant
	30	0.08	0.036	0.107	Not significant
LICI	5	0.28	0.078	0.170	Not significant
	10	0.22	0.085	0.170	Not significant
	15	0.18	0.118	0.202	Not significant
	30	0.31	0.317	0.476	Not significant
ICF	5	0.00036	0.459	0.612	Not significant
	10	0.05	0.623	0.747	Not significant
	15	0.02	0.916	0.978	Not significant
	30	0.000064	0.978	0.978	Not significant

### Short-Interval Intracortical Inhibitory as a Good Predictor of Effectiveness of Intermittent Theta Burst Stimulation-Induced Cortical Plasticity

To assess predictive values for iTBS-induced cortical plasticity, we performed the multiple linear regression analysis with the levels of SICI, LICI, and ICF before iTBS as independent variables, and the dependent variable the after-effects of iTBS-induced cortical plasticity. Results from multicollinearity tests showed that the data met the assumption of collinearity with VIF = 1. Results from stepwise linear regression indicated that the model, with SICI as the only predictor, was significant at the time point of 5 min following iTBS conduction [*F*(1,13) = 10.144, *p* = 0.007, *R^2^* = 0.40] and with beta coefficient = –0.4 for influence of SICI on the iTBS-induced cortical plasticity. In addition, SICI also remained as the only predictor in the models of 10 min [*F*(1,13) = 9.952, *p* = 0.008, *R^2^* = 0.40] and 15 min [*F*(1,13) = 10.733, *p* = 0.006, *R^2^* = 0.41] after conducting iTBS, with beta coefficients = –0.42 and –0.48 for influence of SICI on the iTBS-induced after-effect at 10 and 15 min after stimulation, respectively. *Post hoc* power analysis revealed that coefficients of –0.4, –0.42, and –0.48 could be detected at 0.05 at a power of greater than 0.90.

## Discussion

The current study aimed at providing a direct investigation of the relationship between LTP-like plasticity induced by iTBS in the human motor cortex and the level of GABA or NMDA receptor-mediated activity before stimulation. We used a series of paired-pulse TMS protocols including SICI, LICI, and ICF to assess GABA_A_ receptor-, GABA_B_ receptor-, and glutamate receptor-mediated activity, respectively. Our findings showed that (i) GABA_A_ receptor-mediated activity assessed before stimulation was significantly negatively correlated with LTP-like plasticity induced by iTBS following 5, 10, and 15 min after conduction of stimulation; (ii) there was no significant correlation between LTP-like plasticity induced by iTBS and GABA_B_ receptor- or glutamate receptor-mediated activity before stimulation as assessed by the TMS protocol of LICI and ICF; and (iii) SICI, as the index of GABA_A_ receptor-mediated activity measured before stimulation, is a good predictor of iTBS-induced LTP-like plasticity for a period lasting 15 min following stimulation.

Cortical inhibition is essential for regulating neuronal excitability, and a decrease in local inhibitory signaling is necessary for LTP-like plasticity to occur. In the present study, we found that GABA_A_ receptor-mediated activity before stimulation was significantly negatively correlated with LTP-like plasticity induced by iTBS. This is consistent with previous evidence indicating that reduced GABAergic inhibition can facilitate induction of LTP-like plasticity (Castro-Alamancos et al., 1995; Trepel and Racine, 2000; Chen et al., 2010; Bachtiar and Stagg, 2014). Similar findings have also been observed in patients during chronic stages of stroke recovery that GABA_A_ receptor-mediated activity is reduced compared with healthy controls (Blicher et al., 2009). Furthermore, we also found that SICI measured before stimulation is a good predictor of iTBS-induced LTP-like plasticity for a period lasting 15 min. Our findings might add evidence to the suggestion that SICI in the early recovery phase can be a predictor of LTP-like plasticity in the later recovery stage for patients after stroke (McDonnell et al., 2007; Liuzzi et al., 2014).

In addition to GABA_A_ receptors, GABA_B_ receptors are also thought to have an important role in induction of LTP (Larson and Munkácsy, 2015). In the present study, GABA_B_ receptor-mediated activity before stimulation as assessed by LICI was found not significantly correlated with the after-effects of LTP-like plasticity induced by iTBS. This suggested that the interplay between GABA_B_ receptor-mediated inhibition and plasticity is complex. In addition, metabotropic GABA_B_ receptors have been found to modulate inhibitory neural circuits by mediating long-lasting inhibitory postsynaptic potentials or involve presynaptic autoinhibition of interneurons through GABA_A_ receptors to inhibit GABA release (McDonnell et al., 2006; McDonnell et al., 2007). The former postsynaptic inhibition hyperpolarizes target neurons and reduces LTP-like plasticity, whereas the latter one depolarizes target neurons and results in facilitation of LTP (Mott and Lewis, 1991; Stäubli et al., 1999). In the current study, LICI was used to measure GABA_B_ receptor-mediated effects without distinguishing postsynaptic and presynaptic GABA_B_ effects. On the other hand, McDonnell et al. (2006) and Cash et al. (2010) suggested that these two different GABA_B_ effects should be measured using LICI protocol and induce SICI in the presence of LICI in the human motor cortex. Therefore, although no significant correlation between postsynaptic GABA_B_ effects measured by LICI- and iTBS-induced LTP was found in the present study, future studies need to further investigate the relationship between presynaptic GABA_B_ effects and LTP-like plasticity.

Further, it is not consistent with our expectation that no significant correlation was found between iTBS after-effects and NMDA receptor-mediated activity before iTBS as assessed by ICF. Plentiful studies have shown that the NMDA receptor plays an important role in the development of rapid cortical plastic changes and activation of NMDA receptors is necessary to induce LTP-like plasticity (Hunt and Castillo, 2012; Hasan et al., 2013), while this phenomenon was not confirmed by others (Liao et al., 1995; Selig et al., 1995). Besides, previous work indicated that although LTP is induced by activation of NMDA receptors at synapses, these mechanisms are mediated by AMPA receptors trafficking in postsynaptic neurons (Park et al., 2018; Sumi and Harada, 2020). Further studies need to explore the role the AMPA receptor plays in iTBS-induced LTP-like plasticity and how to separate the effect of the NMDA and AMPA receptors on iTBS-induced after-effects. In addition, it may also be due to the fact that ICF is not an ideal measurement of NMDA receptor-mediated activities as more than one possible neural circuit contribute to ICF (Hanajima et al., 1998). Recent studies combining TMS–MRS methods showed that there was no significant relationship between the ICF protocol with ISI of 12 ms and MRS-glutamate, which questioned ICF as an effective tool to measure the level of NMDA-type glutamate receptor-mediated activity (Stagg et al., 2011; Dyke et al., 2017).

In conclusion, the present study found that GABA_A_ receptor-mediated activity measured before stimulation is negatively correlated with the after-effect of cortical excitability induced by iTBS. SICI, as an index of GABA_A_ receptor-mediated activity measured before stimulation, is a good predictor of iTBS-induced LTP-like plasticity for a period lasting 15 min following stimulation. However, there are several limitations in our study. First, we measured cortical excitability only within 30 min following iTBS protocols; it is unclear what happens after 30 min. In addition, a limited number of TMS pulses were applied to LM1, with only 10 trials for each condition (i.e., SICI, ICF, and LICI). Although we have ensured intraindividual reliability of cortical excitability by performing two sessions of single-pulse TMS with an interval of 5 min, the optimal number of trials was required to reduce interindividual differences in order to make the results more reliable. Furthermore, as a pilot study, the sample size is small and the sex of participants was not very balanced. Although no previous work has reported a sex difference in the iTBS-induced after-effects, further studies are still required to investigate sex differences in iTBS-induced LTP-like plasticity to make our findings more generalized to the entire population.

## Data Availability Statement

The raw data supporting the conclusions of this article will be made available by the authors, without undue reservation.

## Ethics Statement

The studies involving human participants were reviewed and approved by the Ethics Committee of Affiliated Jiangsu Shengze Hospital of Nanjing Medical University. The patients/participants provided their written informed consent to participate in this study.

## Author Contributions

YS, T-FY, and CH had full access to all the data in the study, took responsibility for the integrity of the data and the accuracy of the data analysis, designed the trial, and provided critical revisions to the manuscript. XD wrote the original draft as well as review and editing of the current manuscript. QL performed the data analysis. QL, LQ, and XD performed the study procedures. YS was responsible for the funding acquisition. All authors contributed to the article and approved the submitted version.

## Conflict of Interest

The authors declare that the research was conducted in the absence of any commercial or financial relationships that could be construed as a potential conflict of interest.

## Publisher’s Note

All claims expressed in this article are solely those of the authors and do not necessarily represent those of their affiliated organizations, or those of the publisher, the editors and the reviewers. Any product that may be evaluated in this article, or claim that may be made by its manufacturer, is not guaranteed or endorsed by the publisher.

## Footnotes

1
http://www.chictr.org.cn
